# Teachers as the agent of change for student mental health: the role of teacher care and teacher support in Chinese students’ well-being

**DOI:** 10.3389/fpsyg.2023.1283515

**Published:** 2023-11-28

**Authors:** Tingting Wang

**Affiliations:** School of Music, Shandong Women's University, Jinan, China

**Keywords:** student well-being, mental health, teacher support, teacher care, Chinese students

## Abstract

**Introduction:**

Students in any academic setting typically encounter a wide range of academic problems and challenges, which may endanger their mental health. Since the mental health or well-being of students directly affects their classroom performance, factors helping students overcome their academic challenges need to be widely researched. Considering this, extensive research has been carried out to uncover the personal and situational factors that help students solve their academic problems and improve their well-being. Moreover, due to the invaluable role of teachers in students’ mental conditions, several investigations to date have assessed the impact of teacher communicative behaviors on student well-being.

**Methods:**

To pursue this line of inquiry, the current research assessed the role of teacher support and teacher care in fostering Chinese students’ well-being. To do this, three self-report questionnaires were distributed to 338 undergraduate students. Then, Pearson product–moment and linear regression were performed through IBM SPSS Amos (Version 26) to analyze students’ answers to the self-report questionnaires.

**Results:**

The analysis outcomes divulged a series of strong and positive connections between teacher support, teacher care, and student well-being. The results also showed that teacher support and teacher care can significantly contribute to Chinese students’ well-being.

**Implications:**

Teachers and teacher educators may find these outcomes useful and informative.

## 1 Introduction

The academic success of students is closely tied to their mental or psychological conditions (Cárdenas et al., 2022). This means that absence of psychological disorders like anxiety, stress, and depression notably improves the likelihood of student academic success (Dodd et al., 2021; Akram et al., 2022). Put it another way, students who enjoy an appropriate psychological and mental state are more likely to succeed in the academic environment (Rüppel et al., 2015; Akram et al., 2020; Pan et al., 2023; Wang et al., 2023). This psychological and mental state is technically called well-being (McNaught, 2011; Ross et al., 2020). The term “well-being” generally pertains to “one’s degree of happiness and satisfaction with his/her personal, professional, or social life” (Garg and Rastogi, 2009, p. 43; Wang et al., 2021; Fan and Wang, 2022). With this description in mind, Long et al. (2012) conceptualized student well-being as the level of joy and contentment that students feel in classroom contexts. However, as pointed out by Ruggeri et al. (2020), student well-being is not merely about the existence of positive emotions like happiness and satisfaction. They maintained that the notion of student well-being goes beyond the pursuit of pleasure or happiness and has something to do with students’ functioning in classroom contexts. According to them, student well-being is a sustainable psychological condition that enables students to develop and thrive in academic settings.

The close connection between students’ well-being and their academic success (Dodd et al., 2021; Cárdenas et al., 2022) has encouraged many academics and scholars around the world to scrutinize the positive and negative predictors of student well-being. Many researchers to date (e.g., Zeng et al., 2016; Guerra-Bustamante et al., 2019; Ortiz Alvarado et al., 2019; Kulcar et al., 2023) have inspected students’ personal characteristics to find out their favorable and unfavorable impacts on their subjective or psychological well-being. By the same token, several researchers (e.g., Shoshani and Eldor, 2016; Nguyen et al., 2021; Samsen-Bronsveld et al., 2023) have studied various environmental or situational factors in relation to students’ well-being. Likewise, some scholars (e.g., Braun et al., 2020; Wang et al., 2022; Hu and Wang, 2023; Shephard et al., 2023; Wang and Derakhshan, 2023; Zhi and Wang, 2023) have delved into teacher communicative behaviors to assess their potential influences on students’ well-being. Notwithstanding, the role of some communicative behaviors like teacher care and teacher support in students’ subjective or psychological well-being has remained understudied, which warrants quantitative, qualitative, and mixed methods studies on this topic. Accordingly, in the current quantitative inquiry, we intend to locate the role of teacher care and teacher support in Chinese students’ well-being.

A teacher communicative behavior that may bring substantial changes in students’ psychological and subjective well-being is teacher care (Xie and Derakhshan, 2021; Wang et al., 2022). Teacher care, also called teacher caring, is a communicative activity that helps teachers satisfy their pupils’ psycho-emotional needs and desires (Tosolt, 2010). This communicative activity has been described by Laletas and Reupert (2016) as teachers’ verbal and nonverbal endeavors to address students’ needs through establishing a positive, respectful, and encouraging learning atmosphere. Gabryś-Barker (2016) further referred to this communicative activity as “teachers’ provision of genuine support to students, displaying interest in students’ learning, and being empathetic toward them” (p. 157). As pointed out by Song et al. (2022), verbal and nonverbal caring behaviors empower teachers to develop and maintain effective relationships with students. Effective teacher-student relationships can make some desirable changes in students’ subjective well-being (Graham et al., 2016; Pan et al., 2023), academic motivation (Henry and Thorsen, 2018), and classroom engagement (Zhou, 2021).

Another communicative behavior of teachers that may contribute to students’ psychological and subjective well-being is teacher support (Zheng, 2022). This communicative behavior alludes to “supporting students, helping them solve their problems, and making them feel respected” (Chang and Bangsri, 2020, p. 437; Guo et al., 2023). According to Malecki and Demaray (2003), teacher support can be defined as the “instrumental,” “informational,” “appraisal,” and “emotional” assistance or guidance that teachers offer to students in academic settings. The first form of teacher support, instrumental support, deals with the amount of time and energy teachers spend helping their pupils (Rueger et al., 2008). The second form of teacher support is informational support, which involves helping students understand complex and challenging learning materials (Demaray et al., 2012). The third form of teacher support, known as appraisal support, includes the formative and summative feedback that teachers offer to students in the learning process (Tennant et al., 2015). Emotional support, as the last form of this communicative behavior, captures how teachers care for their students and develop intimate relationships with them (Clark et al., 2020). The instrumental, informational, appraisal, and emotional support that teachers offer in classroom contexts have a desirable influence on students’ academic emotions (Lei et al., 2018), which in turn, ameliorate their mental or psychological conditions (Brandseth et al., 2019).

Considering the interplay between teacher care, teacher support, and student academic emotions, multitudes of inquiries have been carried out on these communicative behaviors and their pedagogical consequences. In fact, several researchers to date (e.g., Feng et al., 2019; Paloș et al., 2020; Derakhshan, 2022; Derakhshan et al., 2022; Wang and Derakhshan, 2023) have attempted to unmask the potential outcomes of teacher care and teacher support in instructional-learning contexts. Nevertheless, the impacts of these communicative behaviors on students’ subjective and psychological well-being have scarcely been scrutinized (Littlecott et al., 2018; Lavy and Naama-Ghanayim, 2020). Thus, whether teacher care and teacher support can influence student well-being is still an open question. In order to shed light on this question, this investigation intends to disclose the role of teacher care and teacher support in Chinese students’ well-being. The following research questions were developed in accordance with the main purposes of the study:

1 Is there any significant association between teacher support, teacher care, and student well-being?
2 To what extent, if any, do teacher support and teacher care significantly predict Chinese students’ well-being?

## 2 Literature review

### 2.1 Teacher support

The variable of teacher support is generally about the degree of assistance, attention, and guidance that students receive within a particular learning environment (Leflot et al., 2011). This variable is made up of three interrelated facets (Lei et al., 2018): “*Structure*,” “*warmth*,” and “*autonomy support*.” The first facet of teacher support, known as structure, concerns the clarity of contingencies and expectations (Jang et al., 2010). The second aspect, which is called warmth, alludes to the closeness and affection between teachers and students (Federici and Skaalvik, 2014). The third facet of this variable is autonomy support, which refers to the provision of multiple choices to students (Su and Reeve, 2011). This aspect of teacher support, as Jang et al. (2016) noted, allows students to take part in the learning process at their own pace. To put in a nutshell, supportive teachers are those who are always available to students during the learning process (Guess and McCane-Bowling, 2016). As demonstrated by earlier inquiries (Samson, 2020; Guo et al., 2021), students who receive adequate and continual support from their teachers are less prone to mental illnesses such as anxiety, stress, and depression.

### 2.2 Teacher care

The concept of care has roots in Aristotle’s rhetorical theory, where he coined the term “goodwill” to describe this emotional labor (McCroskey and Teven, 1999). Goodwill generally includes understanding individuals’ needs and emotions, respecting their personal outlooks, and praising their communicative endeavors (Teven, 2007). Referring to this description, Noddings (2012) conceptualized teacher care as the degree to which teachers recognize their students’ needs and priorities, value their perspectives, and acknowledge their academic efforts. Inspired by Noddings’s definition of teacher care, Ramberg et al. (2019) described this communicative behavior in terms of “understanding,” “empathy,” and “responsiveness.” Understanding, as Ramberg et al. (2019) mentioned, alludes to an individual teacher’s ability to realize students’ academic needs or desires. According to them, empathy relates to an individual teacher’s capacity to comprehend and share his/her students’ feelings and experiences. Finally, responsiveness concerns an individual teacher’s capability to react to students’ demands or problems (Ramberg et al., 2019). As discovered by previous investigations, caring about students’ needs, wants, and preferences promotes their mental health (Lavy and Naama-Ghanayim, 2020) and leads them towards a deeper engagement with the learning environment (Sun, 2021; Zhang, 2021).

### 2.3 Student well-being

Well-being, as defined by the World Health Organization (WHO), is an indicator of mental health measured by an individual’s capacity to manage the daily stress, work productively, and contribute to his or her community (as cited in Li, 2022). In light of this definition, Konu and Rimpelä (2002) conceptualized student well-being as an index of students’ mental health reflected by their ability to regulate their negative emotions, fulfill their learning tasks, and interact with their classmates. Later, De Fraine et al. (2005) referred to student well-being as “the emotional experience shown by the domination of positive emotions and cognition about the learning environments, instructors, and peers” (p. 299). In another effort to characterize student well-being, Ordonez et al. (2011) described this construct by referring to its various dimensions (i.e., psychological well-being and subjective well-being). The first dimension of this construct is psychological well-being that alludes to the absence of mental illnesses (Miller et al., 2013). The second dimension is subjective well-being, which relates to students’ personal and emotional appraisal of their learning experiences (Liu et al., 2016). Previous research on this construct (Rodríguez-Fernández et al., 2018; Ventura-León et al., 2022) has revealed that students with optimum levels of psychological and subjective well-being perform more effectively in academic settings.

### 2.4 Earlier investigations into the interplay between teacher support, teacher care, and student well-being

Despite the interplay between teacher communicative behaviors and students’ mental conditions (Xie and Derakhshan, 2021), few scholars (Zhao et al., 2016; Littlecott et al., 2018; Stallman et al., 2018; Brandseth et al., 2019; Bonneville-Roussy et al., 2020; Lavy and Naama-Ghanayim, 2020; Zheng, 2022; Derakhshan et al., 2023) to date have addressed the role of teachers’ caring and supportive behaviors in students’ subjective or psychological well-being. Stallman et al. (2018), for example, looked into the role of teacher support in college students’ well-being. To do this, 6,195 university college students were asked to complete two web-based questionnaires. The examination of the collected data displayed that teacher support can bring about positive changes in college students’ well-being. In the same vein, Littlecott et al. (2018) probed the function of teacher support in promoting school students’ well-being. To meet this purpose, some semi-structured interviews were performed with a group of secondary school students. The interview outcomes exhibited that supporting students during the learning process can remarkably improve their well-being. Similarly, Brandseth et al. (2019) scrutinized the implications of teachers’ supportive behaviors for students’ mental well-being. To obtain data, two reliable inventories were distributed to 574 high school students. The results pinpointed that high school students’ well-being strictly depends on the emotional and instrumental support they receive in the learning environment. Additionally, to uncover the predicting role of teachers’ caring behaviors, Lavy and Naama-Ghanayim (2020) inspected the impact of teacher care on school students’ well-being. To accomplish this, 676 students were invited to share their views on the interaction between teacher care and student well-being. The analysis of students’ standpoints indicated that teacher care can favorably affect students’ well-being. Likewise, Zheng (2022) evaluated the influence of some teacher communicative behaviors like teacher care on students’ well-being. The study results demonstrated that caring about students can foster their well-being in academic settings.

## 3 Method

### 3.1 Participants

A probability sampling strategy, known as random sampling, was used to choose the participants of the current research. Random sampling is “a subset of sampling techniques in which each sample has an equal probability of being chosen” (Dornyei and Csizer, 2012, p. 81). This sampling technique led to the recruitment of 236 male (70%) and 102 female (30%) students. The participants were all Chinese native speakers whose age ranged from 18 to 34 (*M* = 26, *SD* = 3.4). The participants were undergraduate students chosen from different colleges and universities in China. All participants completed the consent form before they took part in the data collection process.

### 3.2 Instruments

#### 3.2.1 Teacher support scale (TSS)

A modified version of the “*Teacher Support Scale (TSS)*” (Metheny et al., 2008) was used to evaluate students’ attitudes towards their teachers’ supportive behaviors. This scale consists of 21 items, which start with the stem “Most teachers in my school/college.” Instances of items from this scale are: item (4) “*takes the time to help me get better grades*,” item (18) “*supports my goals for the future*,” and item (19) “*will listen if I want to talk about a problem*.” Participants need to rate each of these items on a 5-point, Likert-type scale with answers varying from “strongly agree (scored as 5)” to “strongly disagree (scored as 1).” A Cronbach’s alpha of 0.91 was obtained for the modified version of this scale.

#### 3.2.2 Perceived caring scale (PCS)

The 10-item version of the “*Perceived Caring Scale (PCS)*” (Teven and McCroskey, 1997) was adopted to look into students’ perspectives towards their teachers’ caring behaviors. This measure includes 10 bipolar items. Four items of the PCS are: item (3) “*Self-centered/ Not self-centered*,” item (6) “*Empathetic/ Apathetic*,” item (9) “*Understands how I feel/ Does not understand how I feel*,” and item (10) “*Does not understand how I think/ Understand how I think*.” A reliability index of 0.89 was discovered for the 10-item version of this measure.

#### 3.2.3 Student well-being questionnaire (SWQ)

The psychological and subjective well-being of students was assessed using the “*Student Well-being Questionnaire (SWQ)*” (Butler and Kern, 2016). The SWQ comprises five different subscales, including “*Positive Emotion*” (items 1–3), “*Engagement*” (items 4–6), “*Relationship*” (items 7–9), “*Meaning*” (items 10–12), and “*Accomplishment*” (items 13–15). This inventory is made of 15 closed-ended items, some of which are: item (2) “*How often do you feel positive?*,” item (5) “*To what extent do you feel excited and interested in things?*,” and item (8) “*To what extent have you been feeling loved?*.” The reliability of the SWQ was found to be 0.86.

### 3.3 Data collection procedure

Prior to distributing the valid measures of the variables, some information was delivered to participants regarding the main aims and intentions of the study. All respondents then received some accurate directions on how to fill out the online surveys. Afterwards, the above-mentioned surveys (TSS, PCS, and SWQ) were all translated into Chinese. The questionnaires were then sent to two language experts to verify the accuracy of the translated items. Following that, the translated questionnaires were distributed to participants through QQwenjuan and Wenjuanxing platforms. The QQwenjuan and Wenjuanxing are two professional online survey platforms that help researchers gather a wide range of quantitative data. Using these platforms, the necessary information was collected within 5 days.

### 3.4 Data analysis

To begin with, the Cronbach alpha coefficient was used to compute the instruments’ reliability. The validity of the instruments was subsequently evaluated using the Confirmatory Factor Analysis (CFA). Then, the interplay between teacher support, teacher care, and student well-being was calculated through Pearson product–moment. Following that, linear regression analysis was performed through the IBM SPSS Amos (Version 26) to identify the role of teachers’ caring and supportive behaviors in Chinese students’ well-being. In light of the regression analysis outcomes, a measurement model with standardized estimates was drawn to display the function of teacher support and teacher care in promoting Chinese students’ well-being.

## 4 Results

As mentioned earlier, the reliability and validity of the instruments were calculated before answering the research questions. First, the reliability of scales and subscales was computed through the Cronbach alpha coefficient. The reliability indices are all represented in the table below (Table 1).

**Table 1:** The reliability indices of the instruments (TSS, PCS, SWQ).

Scales and subscales	Cronbach’s alpha coefficient	*N* of items
Student well-being	0.86	15
Positive emotion	0.71	3
Engagement	0.74	3
Relationship	0.79	3
Meaning	0.70	3
Accomplishment	0.76	3
Teacher care	0.89	10
Concern	0.77	3
Empathy	0.71	3
Respect	0.79	4
Teacher support	0.91	21
Interested	0.81	6
Positive regard	0.72	5
Expectation	0.75	5
Accessible	0.76	5

As displayed in Table 1, the three scales and their subscales enjoyed a satisfactory reliability index. Second, CFA was performed to test the validity of the inventories. The results of this multivariate statistical procedure are shown in Table 2.

**Table 2:** The results of CFA.

			Estimate	S.E.	C.R.	*P*
Concern	<−--	Teacher care	0.992	0.050	13.479	0.000
Empathy	<−--	Teacher care	0.949	0.038	24.843	0.000
Respect	<−--	Teacher care	0.679	0.050	13.479	0.000
Interested	<−--	Teacher support	0.386	0.079	4.915	0.000
Positive regard	<−--	Teacher support	0.938	0.048	19.417	0.000
Expectation	<−--	Teacher support	0.993	0.044	24.374	0.000
Accessible	<−--	Teacher support	0.994	0.021	46.914	0.000
Accomplishment	<−--	Students’ well-being	0.991	0.440	5.754	0.000
Meaning	<−--	Students’ well-being	0.985	0.422	5.731	0.000
Relationship	<−--	Students’ well-being	0.976	0.434	5.760	0.000
Engagement	<−--	Students’ well-being	0.987	0.440	5.754	0.000
Positive emotions	<−--	Students’ well-being	0.953	0.427	5.750	0.000
PO1	<−--	Positive emotions	0.992	0.422	5.731	0.000
PO2	<−--	Positive emotions	0.987	0.020	51.904	0.000
PO3	<−--	Positive emotions	0.994	0.021	48.687	0.000
EN1	<−--	Engagement	0.986	0.079	15.979	0.000
EN2	<−--	Engagement	0.994	0.024	41.940	0.000
EN3	<−--	Engagement	0.994	0.022	44.285	0.000
REL1	<−--	Relationship	0.998	0.020	52.902	0.000
REL2	<−--	Relationship	0.989	0.021	46.914	0.000
REL3	<−--	Relationship	0.994	0.020	50.293	0.000
ME1	<−--	Meaning	0.997	0.043	45.354	0.000
ME2	<−--	Meaning	0.997	0.023	43.575	0.000
ME3	<−--	Meaning	0.998	0.023	43.586	0.000
ACO1	<−--	Accomplishment	0.989	0.056	36.426	0.000
ACO2	<−--	Accomplishment	0.915	0.035	26.054	0.000
ACO3	<−--	Accomplishment	0.935	0.035	26.611	0.000
CO1	<−--	Concern	0.998	0.047	34.247	0.000
CO2	<−--	Concern	0.958	0.026	36.496	0.000
CO3	<−--	Concern	0.962	0.026	37.157	0.000
EM3	<−--	Empathy	0.997	0.126	46.214	0.000
EM2	<−--	Empathy	0.914	0.034	26.676	0.000
EM1	<−--	Empathy	0.994	0.035	29.190	0.000
RES3	<−--	Respect	0.987	0.034	26.741	0.000
RES2	<−--	Respect	0.997	0.079	15.979	0.000
RES1	<−--	Respect	0.996	0.077	16.298	0.000
RES4	<−--	Respect	0.995	0.077	15.545	0.000
ACE3	<−--	Accessible	0.997	0.066	27.456	0.000
ACE2	<−--	Accessible	0.954	0.041	23.333	0.000
ACE1	<−--	Accessible	0.947	0.039	23.992	0.000
ACE4	<−--	Accessible	0.997	0.036	28.043	0.000
ACE5	<−--	Accessible	0.998	0.037	27.188	0.000
EX3	<−--	Expectation	0.996	0.048	29.321	0.000
EX2	<−--	Expectation	0.814	0.041	19.882	0.000
EX1	<−--	Expectation	0.898	0.026	34.710	0.000
EX4	<−--	Expectation	0.958	0.023	41.519	0.000
EX5	<−--	Expectation	0.927	0.022	42.844	0.000
PR3	<−--	Positive regard	0.997	0.047	25.416	0.000
PR2	<−--	Positive regard	0.996	0.044	22.479	0.000
PR1	<−--	Positive regard	0.990	0.037	26.982	0.000
PR4	<−--	Positive regard	0.993	0.040	25.056	0.000
PR5	<−--	Positive regard	0.897	0.043	20.776	0.000
IN3	<−--	Interested	0.987	0.049	56.419	0.000
IN2	<−--	Interested	0.997	0.016	64.669	0.000
IN1	<−--	Interested	0.987	0.017	61.771	0.000
IN4	<−--	Interested	0.986	0.017	60.597	0.000
IN5	<−--	Interested	0.997	0.019	52.002	0.000
IN6	<−--	Interested	0.989	0.021	46.401	0.000

**Figure 1:**
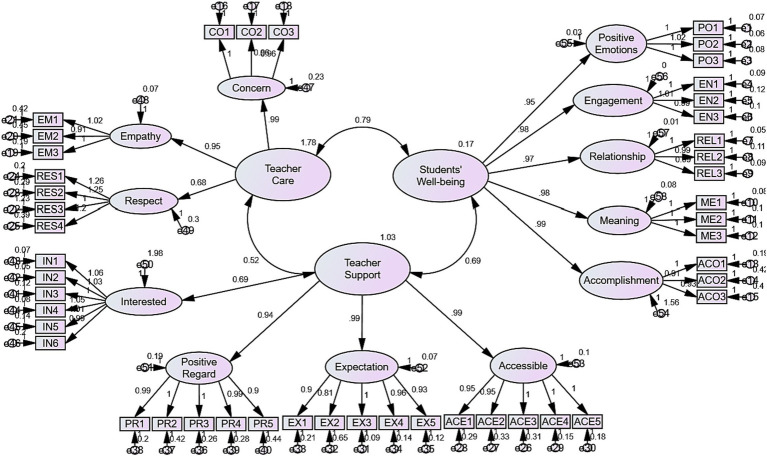
The initial CFA model.

The CFA results revealed that all values are above 0.50, acknowledging the validity of scales and underlying latent constructs. The initial CFA model (Figure 1) was then developed in light of the above-mentioned values.

Afterwards, the quality of the proposed CFA model was checked, and the outcomes are shown in Table 3.

**Table 3:** Evaluation of the CFA model.

			Threshold		
Criteria		Terrible	Acceptable	Excellent	Evaluation
CMIN	3690.486				
DF	974				
CMIN/DF	3.789	> 5	> 3	> 1	Acceptable
RMSEA	0.079	> 0.08	< 0.08	< 0.06	Acceptable
GFI	0.901	< 0.9	> 0.9	> 0.95	Acceptable
CFI	0.901	< 0.9	> 0.9	> 0.95	Acceptable
PNFI	0.791	< 0.5	> 0.5		Acceptable
TLI	0.910	> 0.9	> 0.9	> 0.95	Acceptable

As displayed in table 3, the fit indices, namely “CMIN-DF,” “Goodness-of-Fit Index (GFI),” “Comparative Fit Index (CFI),” “Parsimonious Normed Fit Index (PNFI),” “Tucker–Lewis Index (TLI),” and “Root Mean Square Error of Approximation (RMSEA)” are all within specifications. This means that the proposed CFA model is of acceptable quality. Next, to respond to the first research question, Pearson product–moment was run to measure the interrelationships between teacher support, teacher care, and student well-being. The test outcomes are exhibited in the table below (Table 4).

**Table 4:** Interrelationships between teacher support, teacher care, and student well-being.

		Student well-being	Teacher support	Teacher care
Pearson correlation	Student well-being	1	0.693**	0.794**
	Teacher support	0.693**	1	0.525**
	Teacher care	0.794**	0.525**	1
Sig. (1-tailed)	Student well-being		0.002	0.000
	Teacher support	0.002		0.000
	Teacher care	0.000	0.000	
*N*	Student well-being	338	338	338
	Teacher support	338	338	338
	Teacher care	338	338	338

As shown in the above table, a significant and favorable association (*r* = 0.693, *n* = 338, *p* < 0.002) was discovered between teacher support and student well-being. A similar relationship (*r* = 0.794, *n* = 338, *p* < 0.000) was also found between teacher care and student well-being. The correlation test outcomes also displayed a close, positive (*r* = 0.525, *n* = 338, *p* < 0.000) connection between teacher care and teacher support. Following that, to respond to the second research question, linear regression was done to identify the role of teacher support and teacher care in Chinese students’ well-being. The outcomes of this predictive analysis are presented in Table 5.

**Table 5:** Outcomes of linear regression analysis.

			Estimate	S.E.	C.R.	*P*
Students’ well-being	<−--	Teacher care	0.653	0.015	2.990	0.003
Students’ well-being	<−--	Teacher support	0.651	0.049	5.326	0.000
Concern	<−--	Teacher care	0.942	0.050	13.479	0.000
Empathy	<−--	Teacher care	0.979	0.038	24.843	0.000
Respect	<−--	Teacher care	0.855	0.050	13.479	0.000
Interested	<−--	Teacher support	0.568	0.079	4.915	0.000
Positive regard	<−--	Teacher support	0.910	0.048	19.417	0.000
Expectation	<−--	Teacher support	0.972	0.044	24.374	0.000
Accessible	<−--	Teacher support	0.954	0.021	46.914	0.000
Accomplishment	<−--	Students’ well-being	0.611	0.440	5.754	0.000
Meaning	<−--	Students’ well-being	0.962	0.422	5.731	0.000
Relationship	<−--	Students’ well-being	0.993	0.434	5.760	0.000
Engagement	<−--	Students’ well-being	0.999	0.440	5.754	0.000
Positive emotions	<−--	Students’ well-being	0.984	0.427	5.750	0.000

**Figure 2:**
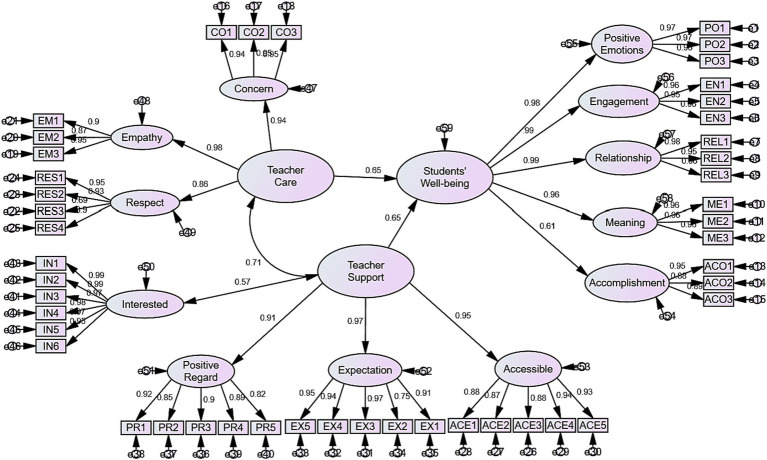
The measurement model.

Finally, in accordance with the regression analysis outcomes, a measurement model (Figure 2) was designed to portray the predicting role of teacher care and teacher support.

As demonstrated in the table and figure above, both teacher support and teacher care predicted about 65% of the change in Chinese students’ well-being. This suggests that these positive communicative behaviors can significantly contribute to Chinese students’ well-being.

## 5 Discussion

The present quantitative inquiry was undertaken with the aim of assessing the interplay between teacher support, teacher care, and student well-being. This inquiry also set out to divulge the role of these communicative behaviors in Chinese students’ well-being. The research outcomes displayed that teacher care and teacher support are closely and positively related to student well-being. The study results also evinced that the caring and supportive behaviors of teachers can bring about desirable changes in Chinese students’ well-being. This implies that supporting students and caring about their academic emotions, needs, and problems can dramatically improve their mental or psychological conditions.

A possible explanation for the strong connection between teacher support and student well-being might be that supporting students to attain their desired learning goals makes students feel happy and satisfied (Lei et al., 2018). These two feelings, as pointed out by Garg and Rastogi (2009), are the main indicators of students’ psychological well-being. This result is in congruence with the outcome of Stallman et al. (2018), who discovered that college students’ well-being is highly correlated with their teachers’ supportive behaviors. This is also aligned with Littlecott et al.’ (2018) findings, which displayed that school students’ well-being is tied to teacher support. Besides, the observed correlation between teacher care and student well-being could be attributed to the role of teachers’ verbal or nonverbal caring behaviors in satisfying students’ academic demands. As noted by Ramberg et al. (2019), addressing students’ academic demands leads them to the optimum level of psychological and subjective well-being. This result is in congruence with that of Zhao et al. (2016), who discovered a close, positive connection between caring and student subjective well-being. This outcome also mirrors Lavy and Naama-Ghanayim’s (2020) results, which indicated school students’ well-being has to do with their instructors caring behaviors. This also seems to be consistent with Zheng’s (2022) outcomes, which demonstrated that students’ well-being is associated with teachers’ communicative behaviors, notably teacher care.

Furthermore, the finding of this investigation about the vital role of teacher support in promoting Chinese students’ well-being can be readily explained by the fact that supporting students throughout the learning process helps teachers ameliorate their pupils’ mental conditions (Brandseth et al., 2019). This backs up the results of Littlecott et al. (2018), who reported that teacher support is one of the main determinants of school students’ well-being. This finding is also in line with that obtained by Stallman et al. (2018), who found that teacher support is an important source of students’ well-being in academic environments. In addition, the current study’s outcome on the pivotal function of teacher care in enhancing Chinese students’ well-being may have something to do with the impact of teachers’ relational behaviors on students’ subjective and psychological well-being (Houser and Hosek, 2018). As put by Houser and Hosek (2018), the relational behaviors used by teachers in educational environments can significantly promote students’ subjective and psychological well-being. This further supports the results of Zhao et al. (2016), who found that caring and resilience had a direct impact on students’ subjective well-being. This result also accords with that discovered by Lavy and Naama-Ghanayim (2020), who reported that teacher caring behaviors can significantly contribute to school student’ well-being. This further reflects Zheng’s (2022) findings, which revealed that teacher care plays a critical role in increasing students’ well-being.

In the end, it is worth mentioning that the current inquiry suffers from some limitations and shortcomings, which justify future empirical investigations into this topic. The main limitation of this investigation concerns the scope of the study, which was limited to the Chinese educational environment. To enhance the transferability of findings, future researchers should employ a multinational or a cross-cultural design to evaluate the role of these communicative behaviors in students’ well-being Another shortcoming of this research is about the data-gathering instrument. This research only used self-report questionnaires to assess participants’ beliefs and attitudes. In the future, scholars can make use of other instruments like observations, narrative writings, and interviews to attain more comprehensive outcomes. The last shortcoming of this study is related to the ignorance of the contextual variables, which may somehow influence the interplay between the variables under investigation. Future studies on this topic can address the influence of situational variables such as age, gender, and educational environment on the association between teacher support, teacher care, and student well-being.

## 6 Conclusions and implications

In this investigation, the primary objective was to evaluate the connections between teacher support, teacher care, and student well-being. Another goal of this investigation was to determine the role of teacher support and teacher care in promoting Chinese students’ well-being. By drawing on the results obtained in the study, it can be argued that teachers’ communicative behaviors like teacher support and teacher care are of paramount importance in improving the well-being of Chinese students. Put it another way, Chinese students’ perceptions of their teachers’ caring and supportive behaviors can largely influence their psychological and subjective well-being. This may be instructive for all in-service teachers who seek to bring about positive changes in their students’ well-being. In order to promote the psychological and subjective well-being of students, teachers must continuously support them in the learning process. For this purpose, they are also required to care about their students’ needs, wants, and emotions. Teacher educators may also find the study outcomes momentous and hold some workshops and training courses on teacher communicative behaviors and their influences on students’ well-being.

## Data availability statement

The original contributions presented in the study are included in the article/supplementary material, further inquiries can be directed to the corresponding author/s.

## Ethics statement

The studies involving humans were approved by the Shandong Women’s University Academic Ethics Committee. The studies were conducted in accordance with the local legislation and institutional requirements. The participants provided their written informed consent to participate in this study.

## Author contributions

TW: Data curation, Formal Analysis, Validation, Writing – original draft,Writing – review & editing.
